# Mass balance of the Greenland and Antarctic ice sheets from the 1970s to 2023

**DOI:** 10.1038/s41597-026-08088-0

**Published:** 2026-09-16

**Authors:** Inès N. Otosaka, Andrew Shepherd, Charles Amory, Martin Horwath, Erik R. Ivins, Michalea D. King, Sophie Nowicki, Anthony J. Payne, Eric Rignot, Louise Sandberg Sørensen, Nicole-Jeanne Schlegel, Karen M. Simon, Benjamin E. Smith, Tyler C. Sutterley, Michiel R. van den Broeke, Isabella Velicogna, Geruo A, Cécile Agosta, Pavel Ditmar, Thorben Döhne, Marcus E. Engdahl, Xavier Fettweis, Rene Forsberg, Alex S. Gardner, Linda Gilbert, Heiko Goelzer, Noel Gourmelen, Andreas Groh, Nicolaj Hansen, Christopher Harig, Veit Helm, Shfaqat Abbas Khan, Christoph Kittel, Peter L. Langen, Mathias Larsen, Bryant D. Loomis, Malcolm McMillan, Brooke Medley, Daniele Melini, Ruth H. Mottram, Alan Muir, Johan Nilsson, Brice Noël, Mark E. Pattle, Mònica Roca i Aparici, Ingo Sasgen, Himanshu V. Save, Bernd Scheuchl, Ernst J. O. Schrama, Ludwig Schröder, Ki-Weon Seo, Sebastian B. Simonsen, Thomas Slater, Giorgio Spada, Bramha Dutt Vishwakarma, Nander Wever, David N. Wiese, Bert Wouters

**Affiliations:** 1 Centre for Polar Observation and Modelling, Department of Geography and Environmental Science, Northumbria University, Newcastle-upon-Tyne, UK; 2 Institut des Géosciences de l’Environnement, University Grenoble Alpes, CNRS, Grenoble, France; 3 Institute of Planetary Geodesy, TUD Dresden University of Technology, Dresden, Germany; 4 Jet Propulsion Laboratory, California Institute of Technology, Pasadena, USA; 5 Polar Science Center, University of Washington Applied Physics Laboratory, Seattle, WA USA; 6 Department of Geology and RENEW Institute, University at Buffalo, Buffalo, USA; 7 Department of Earth, Ocean and Ecological Sciences, University of Liverpool, Liverpool, UK; 8 Earth System Science, University of California Irvine, Irvine, USA; 9 DTU Space, Technical University of Denmark, Kgs, Lyngby, Denmark; 10 NOAA/OAR, Geophysical Fluid Dynamics Laboratory, Princeton, NJ USA; 11 Canadian Geodetic Survey, Ottawa, Canada; 12 Institute for Marine and Atmospheric Research Utrecht, Utrecht University, Utrecht, The Netherlands; 13 Laboratoire des Sciences du Climat et de l’Environnement, LSCE-IPSL, CEA, CNRS, UVSQ, UMR8212, Université Paris Saclay, Gif-sur-Yvette, France; 14 Department of Geoscience and Remote Sensing, Delft University of Technology, Delft, The Netherlands; 15 Climate Action, Sustainability and Science Department, European Space Agency, Frascati, Italy; 16 Laboratory of Climatology, Department of Geography, SPHERES research unit, University of Liège, Liège, Belgium; 17 MSSL, University College London, London, UK; 18 NORCE Research, Bjerknes Centre for Climate Research, Bergen, Norway; 19 School of Geosciences, University of Edinburgh, Edinburgh, UK; 20 National Centre for Climate Research, Danish Meteorological Institute, Copenhagen, Denmark; 21 Department of Geosciences, University of Arizona, Tucson, USA; 22 Glaciology, Alfred-Wegener-Institut Helmholtz-Zentrum für Polar- und Meeresforschung, Bremerhaven, Germany; 23 Physical Geography Research Group, Department of Geography, Vrije Universiteit Brussel, Brussels, Belgium; 24 Department of Environmental Science, iClimate, Aarhus University, Roskilde, Denmark; 25 Geodesy and Geophysics Laboratory, NASA GSFC, Greenbelt, USA; 26 UK Centre for Polar Observation and Modelling, Centre of Excellence in Environmental Data Science, Lancaster Environment Centre, Lancaster University, Lancaster, UK; 27 Earth Sciences Division, NASA Goddard Space Flight Center, Greenbelt, USA; 28 Istituto Nazionale di Geofisica e Vulcanologia, Roma, Italy; 29 Space and Climate Physics, University College London, London, UK; 30 Department of Earth Science, Uppsala University, Uppsala, Sweden; 31 isardSAT, Guildford, UK; 32 isardSAT, Barcelona, Catalonia; 33 Institute of Geography, University of Augsburg, Augsburg, Germany; 34 Center for Space Research, The University of Texas at Austin, Austin, USA; 35 Faculty of Aerospace Engineering, Delft University of Technology, Delft, The Netherlands; 36 Seoul National University, Seoul, South Korea; 37 Dipartimento di Fisica e Astronomia “Augusto Righi”, Università di Bologna, Bologna, Italy; 38 Interdisciplinary Centre for Water Research, Indian Institute of Science, Bengaluru, India; 39 Centre for Earth Sciences, Indian Institute of Science, Bengaluru, India; 40 WSL Institute for Snow and Avalanche Research SLF, Davos, Switzerland

## Abstract

The Greenland and Antarctic ice sheets are major drivers of global mean sea level rise and are predicted to continue to do so in the future. However, they also represent the largest source of uncertainty in projections of future sea level rise making robust estimates of observed ice sheet mass changes critical. Here, we compare and combine 42 independent estimates of ice sheet mass balance derived from satellite observations of temporal changes in ice sheet flow, volume, and gravitational attraction to determine the ice sheet mass balance from 1972 (Greenland) and 1979 (Antarctica) until 2023. We then use regional climate models to partition the total mass balance into contributions associated with surface mass balance and ice dynamical imbalance. The ice sheets lost 11,309 ± 565 billion tonnes of ice between 1979 and 2023, with glacier dynamical imbalance driving 84% of the ice loss and surface mass balance the remainder. This dataset can be used to track the contribution of the ice sheets to sea level rise and constrain projections of future sea level rise.

## Background & Summary

Since the 1990s, the pace of ice loss from the Antarctic and Greenland ice sheets has accelerated, contributing to the increase in global mean sea level rise^1^, with the ice sheets now representing almost a quarter of global sea level rise^2^. As ice losses from the ice sheets are predicted to further increase over the coming decades and centuries^3^, quantifying ice sheet mass balance – the rate of change in ice sheet mass – is thus critical to assess the ice sheets’ contribution to rising sea levels and the risks associated for coastal communities around the world^4,5^. The ice sheets also dominate uncertainties in future sea level rise, and high-end sea-level rise projections increase by a factor 2.6 by 2150 if ice sheet instability processes are included^5^. Maintaining the long-term observational record of ice sheet mass balance is a critical step towards improved ice sheet model skill^6,7^ and realistic projections of sea level rise^8,9^.

Fluctuations in ice sheet mass are the results of ice dynamical, surface mass balance (SMB), and basal mass balance processes. While ice sheets gain mass primarily from snowfall accumulation, they lose mass through several processes, including solid ice discharge at the grounding line, melting at the bed and at the ice-ocean interface, and through surface meltwater runoff, sublimation, evaporation, and blowing snow erosion. Ice velocity measured by radar and optical imaging sensors, volume changes measured by radar and laser altimeters, and Earth’s gravity field variations measured by microwave ranging interferometers are the key satellite records used to monitor the state of the ice sheets^10^. When combined with models of SMB, firn processes, basal mass balance, and glacial isostatic adjustment (GIA), they provide estimates of ice sheet mass balance. In the input-output method^11–13^ (also called mass budget method), mass balance within glacier drainage basins is derived from combining solid ice discharge estimates (derived from ice velocity measurements and estimates of ice thickness at the grounding line) and regional climate model estimates of the individual SMB components^14,15^. In marine-terminating glacier basins, ice discharge is calculated at flux gates defined in the vicinity of the grounding line, either at a fixed distance upstream from the grounding line, inland of the most retreated grounding line location recorded or where airborne measurements of ice thickness are available. The grounding line flux is then calculated by combining ice velocity and ice thickness measurements and is adjusted by adding the average SMB for the area between the flux gate and ice front. A correction is further applied to time-series of grounding line flux to account for temporal changes in ice thickness using surface elevation change measurements. Finally, mass balance is derived by differencing this ice discharge estimate and the basin integrated SMB. Altimetry-derived mass balance estimates^16–18^ are derived by correcting ice sheet volume changes (estimated from repeated elevation surveys) for firn densification processes or by prescribing the density at which the volume changes are occurring with the aid of an external firn density model^19^. Gravimetry-derived mass balance estimates^20–22^ (computed using global spherical harmonic solutions or spatially discrete mass concentration units) are corrected for long-term GIA trends and mass change redistribution processes occurring outside the ice sheets^23,24^.

With more than 50 published estimates of ice sheet mass balance computed using different satellite measurements, geophysical corrections, and ancillary datasets, now available, it is critical to assess differences between estimates, understand the associated uncertainties, and provide a robust combined dataset that can be used by the wider scientific community. This dataset builds on the most recent Ice Sheet Mass Balance Inter-comparison Exercise (IMBIE) assessments for the Antarctic and Greenland ice sheets, which reported a combined contribution of 21.0. ± 1.9 mm to GMSL between 1992 and 2020^2^. Here, we present new consensus estimates of ice sheet mass balance of the Greenland and Antarctic ice sheets for the periods 1972 to 2023 and 1979 to 2023, respectively. Our assessment only accounts for ice sheet mass change that contributes to sea level and does not address the ice loss from ice shelf thinning^25,26^, ice shelf retreat^27^ or ice retreat for ice grounded below sea level^28^. In addition to ice sheet net mass balance, our dataset also includes the partitioning of ice sheet mass trends into SMB and ice dynamical processes, providing key information on the drivers of observed mass changes.

## Methods

### Input data

We use ice sheet mass balance estimates derived using data from 27 satellite missions spanning the years 1972 to 2023 plus a range of GIA and SMB models using common definitions^29,30^ of the Greenland, Antarctic, Antarctic Peninsula, East Antarctic, and West Antarctic ice sheet boundaries (GrIS, AIS, APIS, EAIS, WAIS) (Table 1). In this new assessment, we make use of early satellite velocity measurements going back to the start of the Landsat historical archive in 1972 for Greenland and in 1979 for Antarctica when spatial coverage was sufficient to interpolate or scale these partial velocity surveys to estimate ice discharge at the continental scale. In years with no or poor velocity measurements (for instance between Landsat-1/2 and Landsat-4/5), ice discharge is linearly interpolated, and its uncertainty is increased proportionally to the time span since the closest available velocity estimate. There are 23 estimates of Greenland mass balance altogether comprising 6 altimetry estimates, 14 gravimetry estimates, and 3 input-output estimates. Over Antarctica, we include 19 estimates altogether with 5 of those estimates derived from altimetry, 13 from gravimetry, and 1 from the input-output method. Each estimate is associated with a published study that describes the data and methods used to derive it, although in many cases the estimates have been updated to extend their temporal coverage beyond that of the original study (Table 2).

**Table 1:** Synthesis of satellite datasets, GIA, and SMB models used to derive the individual estimates of ice sheet mass balance from the input-output method (IOM), satellite altimetry (ALT), and satellite gravimetry (GMB) included in this study.

**Table 2:** References of the datasets and methods, employed by participants of the input-output (IOM), altimetry (ALT) and gravimetry (GMB) experiment groups.

	Participant name	Spatial Coverage	Original Temporal Coverage	Extended Temporal Coverage for this assessment	Email address of main point of contact
**IOM**	King^31^	GrIS	1985 to 2018	1985 to 2023	michalea@uw.edu
	Mankoff^47^	GrIS	1840 to 2021	1986 to 2023	mankoff@gmail.com
	Mouginot^13^	GrIS	1972 to 2018	1972 to	erignot@uci.edu
	Rignot^12^	AIS	1979 to 2017	1979 to 2021	erignot@uci.edu
**ALT**	Gourmelen^48^	GrIS	2010 to 2016	2011 to 2018	noel.gourmelen@ed.ac.uk
	Helm^49^	GrIS, AIS, APIS, EAIS, WAIS	2011 to 2013	1992 to 2023	veit.helm@awi.de
	Khan^50^	GrIS	2011 to 2020	1995 to 2023	abbas@space.dtu.dk
	Nilsson^51^	GrIS, AIS, APIS, EAIS, WAIS	1985 to 2020	1992 to 2023 (GrIS)1992 to 2020 (AIS)	johann.n.nilsson@gep.uu.se
	Sørensen^52^	GrIS	2003 to 2008	2018 to 2021	slss@space.dtu.dk
	Schröder^53^	AIS, APIS, EAIS, WAIS	1978 to 2017	1992 to 2017	ludwig.schroeder@gmx.net
	Shepherd^17^	AIS, APIS, EAIS, WAIS	1992 to 2017	1992 to 2023	andrew.shepherd@northumbria.ac.uk
	Simonsen^54^	GrIS	1992 to 2019	1992 to 2019	ssim@dtu.dk
	Smith^18^	GrIS, AIS, APIS, EAIS, WAIS	2003 to 2019	2003 to 2019	besmith@uw.edu
**GMB**	Ditmar^55^	GrIS	2002 to 2023	2002 to 2023	P.G.Ditmar@tudelft.nl
	Döhne^56^	GrIS, AIS, APIS, EAIS, WAIS	2002 to 2020	2002 to 2023	martin.horwath@tu-dresden.de
	Forsberg^57^	GrIS, AIS, APIS, EAIS, WAIS	2002 to 2015	2015 to 2023	rf@space.dtu.dk
	Groh^58^	GrIS, AIS, APIS, EAIS, WAIS	2002 to 2020	2002 to 2023	groh.andreas@gmx.de
	Harig^59^	GrIS, AIS, APIS, EAIS, WAIS	2002 to 2011	2002 to 2023	charig@arizona.edu
	Loomis^60^	GrIS, AIS, APIS, EAIS, WAIS	2003 to 2017	2002 to 2023	bryant.d.loomis@nasa.gov
	Sasgen^61^	GrIS, AIS, APIS, EAIS, WAIS	2003 to 2009	2002 to 2023	ingo.sasgen@awi.de
	Save^62^	GrIS, AIS, APIS, EAIS, WAIS	2003 to 2015	2002 to 2023	save@csr.utexas.edu
	Schrama^63^	GrIS, AIS, APIS, EAIS, WAIS	2003 to 2013	2002 to 2023	e.j.o.schrama@tudelft.nl
	Seo^64^	GrIS, AIS, APIS, EAIS, WAIS	2003 to 2014	2002 to 2023	seokiweon@snu.ac.kr
	Sutterley^65^	GrIS	2002 to 2019	2002 to 2023	tsutterl@uw.edu
	Velicogna^21^	AIS, APIS, EAIS, WAIS	2002 to 2019	2002 to 2021	isabella@uci.edu
	Vishwakarma^66^	GrIS, AIS	2004 to 2013	2018 to 2021	bramha@iisc.ac.in
	Wiese^67^	GrIS, AIS, APIS, EAIS, WAIS	2003 to 2013	2002 to 2023	david.n.wiese@jpl.nasa.gov
	Wouters^68^	GrIS, AIS, APIS, EAIS, WAIS	2002 to 2016	2002 to 2023	Bert.Wouters@tudelft.nl

Compared to the original publication, most datasets have been updated as part of this new reconciled mass balance assessment; both the original temporal coverage and the extended temporal coverage are indicated. To get access to the most recent version of specific individual dataset, please get in touch with the corresponding main point of contact.

### Standardisation of input datasets

**Fig. 1:**
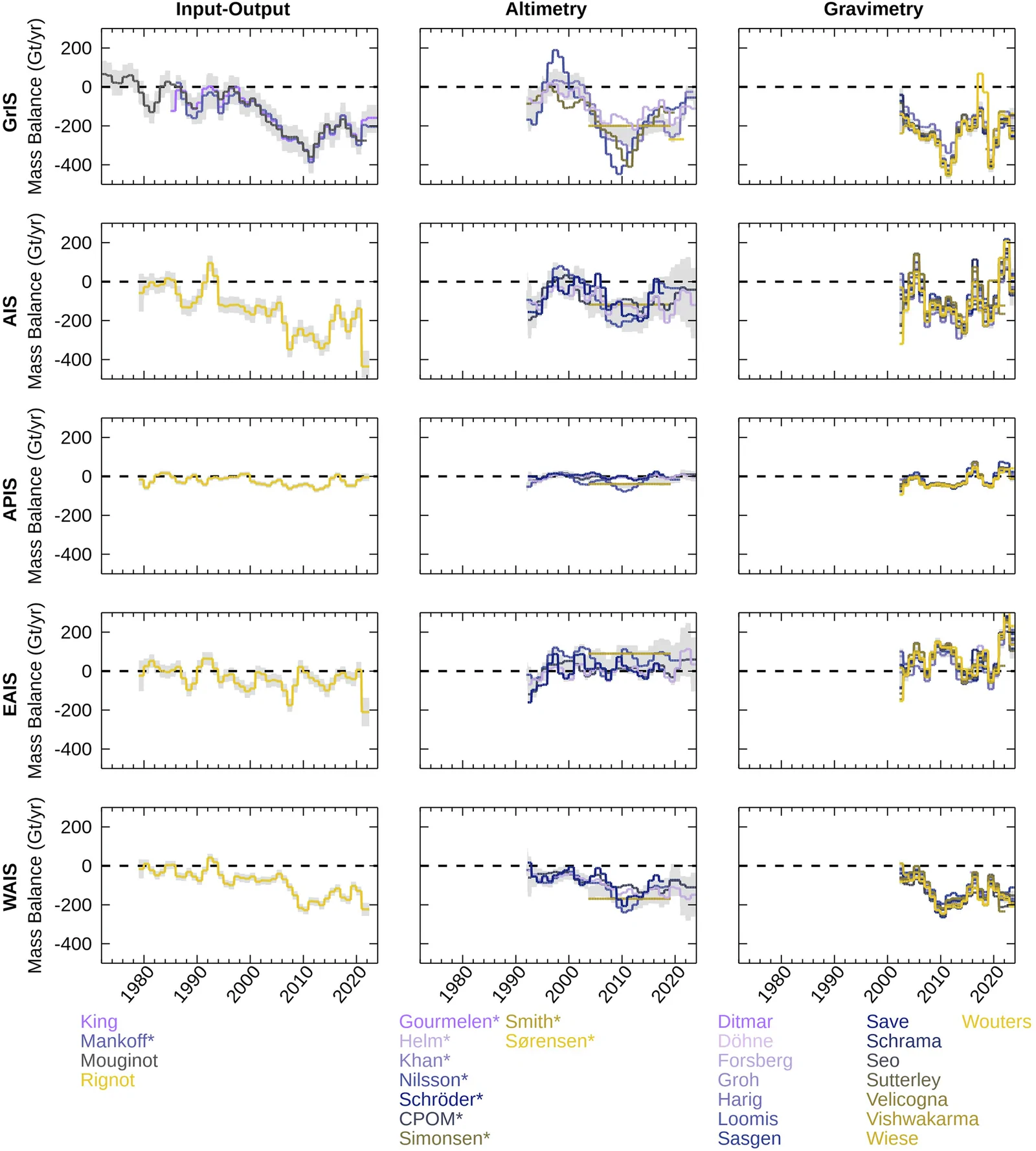
Standardised rates of ice sheet mass balance from the input-output, altimetry, and gravimetry individual estimates over the GrIS, AIS, APIS, EAIS, and WAIS included in this study. The grey shading shows the estimated uncertainty of the aggregated time-series per group. The individual estimates that do not include the peripheral glaciers and ice caps are indicated with an asterisk.

First, we standardise all input datasets as time-series of rates of ice sheet mass change posted at regular monthly intervals. For input datasets originally posted as relative mass change, we estimate the rate of mass change in each month of the record by fitting a linear trend to the temporal mass changes falling within a 36-month sliding window centred around the given month using a weighted least-squares approach (Fig. 1). For the first and last 18 months of the time-series, we reduce the width of the sliding window maintaining its symmetry, except for the very first and last six months for which we use the mean rate calculated over a 12-month period. The error on the standardised mass balance time-series is computed as the sum in quadrature of the standard error of the linear regression and the mean of the input errors divided by the duration of the sliding window. In this scheme, the only difference with the previous standardisation procedure^2^ is that we now divide the mean of the input errors by the duration of the sliding window.

### Accounting for the Ice Sheets’ Peripheral Glaciers and Ice Caps

We correct the input datasets that do not originally account for the ice sheets’ peripheral glaciers and ice caps (GICs) mass changes. While all our gravimetry estimates account for both the main ice sheets and their periphery (here we assume that this does not include the Canadian Arctic peripheral glaciers), this is not the case of our altimetry estimates and one of our input-output estimates. To correct for the omission of Greenland’s GICs, we aggregate two estimates from the input-output method^13,31^ and one from DEM differencing^32^. The two input-output estimates are both derived based on the assumption that GICs mass changes are predominately SMB-driven and are in good agreement with a root-mean-square deviation (RMSD) of 6.9 Gt yr^−1^ and correlation (r^2^) of 0.62 during their overlap period (1985–2018). On the other hand, the rate of mass loss derived from DEM-differencing is 44% and 55% higher than that the two input-output estimates over the period 2002–2018, respectively. The discharge of the GICs has been independently estimated^33,34^ to be of the order of ~2–5 Gt yr^−1^, which is not sufficient to reconcile the SMB-driven GICs estimates with the DEM differencing estimate. We standardise and aggregate these three GICs mass balance time-series, showing that over the period 1972–2023, Greenland’s GICs lost mass at a rate of 8.0 ± 0.5 Gt yr^−1^, which represents around 7% of the ice sheet mass loss. Our aggregated estimate further shows that the GICs started to lose mass in the 1990s at a rate of 4.4 ± 0.8 Gt yr^−1^, followed by a fourfold increase in mass loss, reaching a rate of 18.9 ± 1.3 Gt yr^−1^ in the 2010s. In Antarctica, there are fewer estimates available of the GICs and large disagreement between those estimates. One input-output estimate find the Antarctic periphery in balance over the period 1979–2017^12^, while the DEM differencing method estimates the Antarctic GICs to be losing mass at a rate of 20.9 ± 4.9 Gt yr^−1^ over the period 2010–2018^32^, and an independent CryoSat-2 swath altimetry estimate records rate of mass loss 3.5 ± 0.4 Gt yr^−1^ over the period 2010–2020^35^. We thus do not attempt to correct the altimetry time-series for their omission of the GICs mass changes in Antarctica.

### Aggregation of Ice Sheet Mass Balance Estimates

To produce a reconciled estimate of ice sheet mass balance, we use a similar approach than described in our previous mass balance assessment^2^. Here we briefly summarise this approach. First, we aggregate our time-series of standardised ice sheet mass trends within the altimetry, gravimetry, and input-output groups separately. To do this, we calculated the error weighted average of the individual monthly rates of ice sheet mass change falling within the same satellite class. The only difference with our previous aggregation scheme^2^ is that the weighting applied is the inverse square of the input error instead of the inverse of the error. The error on the technique aggregated time-series is computed at each epoch as the sum in quadrature of the errors of the contributing time-series divided by the square root of the number of estimates available. We then combine the resulting three aggregated time-series into a single reconciled time-series by taking the error-weighted average across the three techniques, using the inverse square of the associated errors as weighting factor, and estimate the associated error as the sum in quadrature of the aggregated time-series errors divided by the square root of the number of techniques available. Finally, we integrate our reconciled time-series of mass trends to generate a time-series of cumulative ice sheet mass change and estimate the cumulative errors as the root sum square of errors, divided by 12.

### Partitioning of Mass Trends into SMB and Ice Dynamics

To determine the proportion of mass loss due to surface and ice dynamical processes, we compute the contemporaneous trend in ice sheet SMB and remove this from our reconciled total mass change estimates. Because direct observations of ice sheet SMB are too scarce to provide full temporal and spatial coverage^36,37^, large scale estimates are taken from atmospheric models which are evaluated with available *in situ* observations. Previous studies showed that finer-spatial-resolution regional climate models produce consistent results due to their ability to capture localised changes and resolve the full extent of the ablation zone^38^. Therefore, we combine estimates of ice sheet SMB from 4 regional climate models in Antarctica and 3 in Greenland (Table 3). When available, the uncertainty of individual SMB models is derived from model-observation comparisons. SMB error from MAR and HIRMHAM were computed using an error function based on a piecewise linear fit, calculated from the root-mean-square error (RMSE) of the 80 percent of model-observation comparison pairs that best match^14,15^. For the GSFC-FDM, SMB error was estimated in relative terms and is reported as the median of the absolute bias between each *in-situ* measurements and modelled estimates of SMB, resulting in a typical relative bias for the GrIS and AIS of 14% and 23%, respectively^39^. Finally, a conservative 20% error estimate was applied to RACMO SMB products, based on previous comparisons with *in-situ* observations of SMB^13^ and satellite observations of annual surface melt^40^.

**Table 3:** Summary of SMB models used to partition ice sheet mass balance.

	Participant	Model	Atmospheric Forcing	Spatial resolution (km)	Temporal coverage	Temporal resolution
**AIS**	Kittel-Amory-Agosta^69^	MAR v3.12.1	ERA5	35	1979–2023	Monthly
	Hansen^70^	HIRHAM5	ERA-interim (1980–2018) & ERA5 (2019–2021)	12.5	1980–2021	Monthly
	Medley^39^	GSFC-FDMv1.2.1	MERRA-2	12.5	1980–2021	5-day
	Van Wessem^71^	RACMO v2.3p2	ERA5	27	1979–2021	Monthly
**GrIS**	Fettweis^72^	MAR v3.12.1	ERA5	10	1950–2023	Monthly
	Hansen^73^	HIRHAM5	ERA-interim (1980–2018) & ERA5 (2019–2021)	5.5	1980–2021	Monthly
	Noël^74^	RACMO v2.3p2	ERA5	1	1958–2023	Monthly

First, we aggregate estimates from the individual SMB models onto a common monthly temporal sampling before averaging into a single combined estimate. We estimate the uncertainty on the combined SMB time series as the sum in quadrature of the individual uncertainty estimates divided by the number of independent estimates used per epoch. For each ice sheet, we then resample these to an annual temporal resolution and compute the anomaly from the SMB combined estimate with respect to reference periods of 1979–2008 and 1960–1990 for Antarctica and Greenland, respectively – approximately 30-year periods which correspond to a period when each ice sheet was in approximate balance^12,41^. Finally, we compute rates of ice sheet dynamic imbalance anomaly as the difference between rates of total ice sheet mass balance and SMB anomaly. We estimate the uncertainty on the rate of ice sheet dynamic imbalance anomaly by taking the root sum square of the estimated uncertainty on the rate of total mass balance and surface mass change anomaly at each epoch. Finally, we integrate monthly rates of each component to obtain estimates of cumulative mass change, surface mass change, and ice dynamic mass change. We estimate the cumulative errors as the root sum square of annual errors, assuming they are not correlated over time.

## Data Records

Our dataset is available at https://doi.org/10.5285/128c5e33-5224-4197-82f0-19dcc95b80a0^42^.

The data consists of a single reconciled estimate of ice sheet mass balance covering the period 1^st^ July 1971 to 31^st^ December 2023 for the GrIS and the period 1^st^ January 1979 to 31^st^ December 2023 for the AIS, APIS, EAIS, and WAIS. Two CSV files are provided for each ice sheet region, one with the data provided in Gigatons (Gt) and one with the data provided in equivalent sea level contribution in millimetres (mm) (assuming that 360 Gt of ice lost is equivalent to 1 mm sea level rise), for a total of 10 files. Files with the data in Gt and mm follow the naming convention: imbie3_*[ice sheet name]*_partitioned_Gt.csv and imbie3_*[ice sheet name]*_partitioned_mm.csv, respectively. The corresponding ice sheet names are: ‘greenland’, ‘antarctica’, ‘antarctic_peninsula’, ‘east_antarctica’, and ‘west_antarctica’. These files contain both annual rates and cumulative anomalies in SMB, dynamics, and total mass change with their corresponding uncertainties.

## Data Overview

**Fig. 2:**
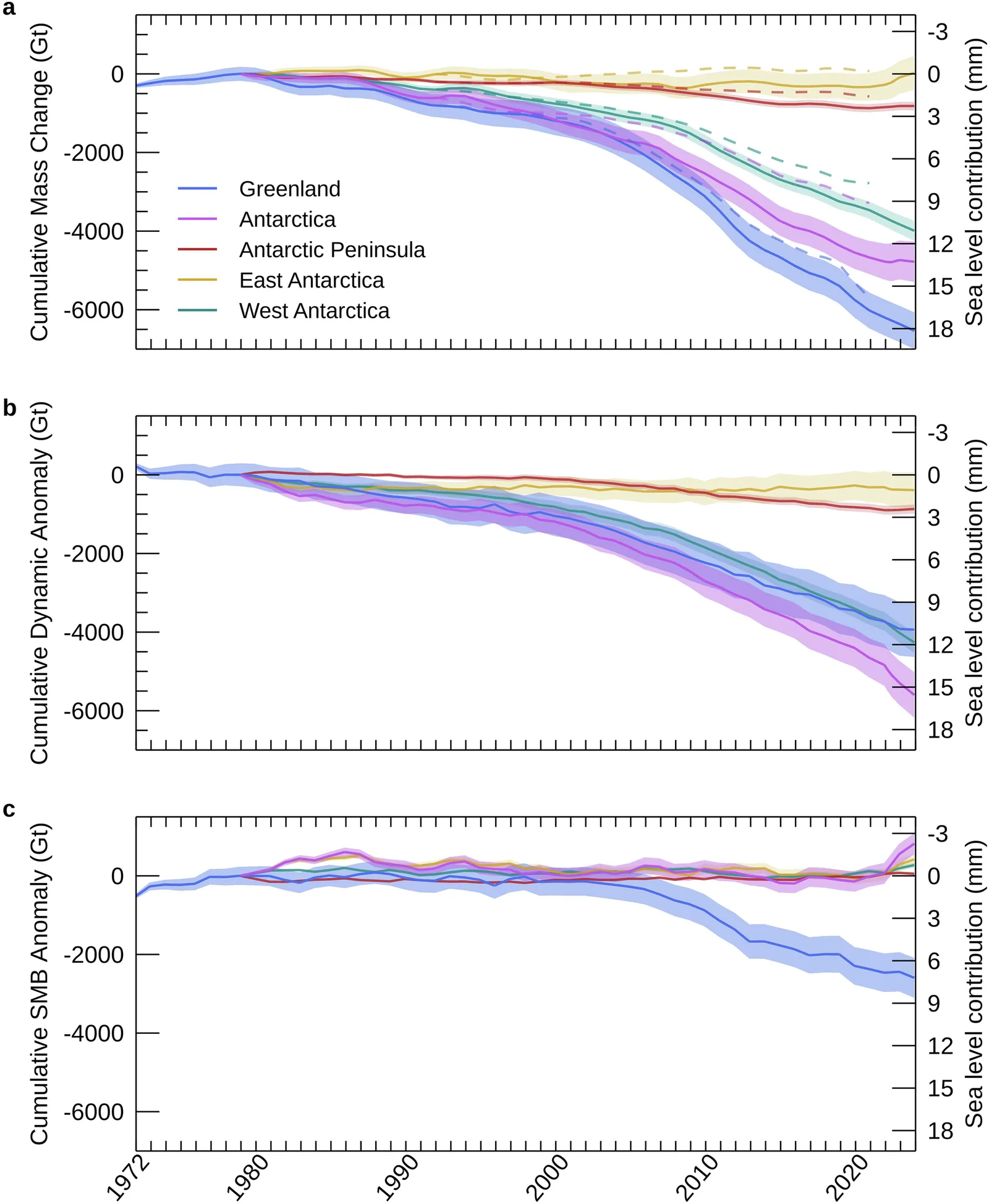
Cumulative ice sheet mass change and anomalies in SMB and dynamics. The shadings represent the associated uncertainties. The dashed lines show the results from a previous ice sheet mass balance assessment^2^.

Our dataset shows that Antarctic mass loss continues to be dominated by ice discharge from West Antarctica where the mass loss signal is strongest (Fig. 2) – rising in every decade of our record from 29 ± 7 Gt yr^−1^ in the 1980s to 163 ± 12 Gt yr^−1^ in the 2010s (Table 4). In the last four years of our survey, ice discharge in West Antarctica further increased by 38% but this was accompanied by an increase in SMB, offsetting this increase in ice discharge. At the Antarctic Peninsula the increase in losses since the early 2000s that is generally associated with ice-shelf collapse^43,44^ led to a rate of mass loss of 32 ± 5 Gt yr^−1^ in the 2000s, four times that of the 1990s. The Antarctic Peninsula lost mass at a similar pace in the 2010s, however during the last four years of our survey (2020 to 2023), the Peninsula was close to a state of balance due to increased snowfall accumulation (22 ± 2 Gt yr^−1^) and reduced ice discharge (11 ± 11 Gt yr^−1^). In East Antarctica, the average 45-year mass trend is 1 ± 9 Gt yr^−1^, however, record high snowfall precipitation in 2022 and 2023 led to a total positive mass gain of 92 ± 63 Gt yr^−1^ during the last four years of our record. This recent mass gain in East Antarctica counterbalanced the sustained mass loss in West Antarctica and as a result Antarctica’s rate of mass loss was more than three times smaller during the period 2020–2023 than in the 2010s. In all, the Antarctic Ice Sheet lost 4,780 ± 513 Gt of ice between 1979 and 2023, raising the global sea level by 13.3 ± 1.4 mm, with all of Antarctica’s ice losses caused by ice dynamics. On the other hand, Greenland was close to a state of balance in the 1970s but started to lose mass in the following decades, with rates of mass loss of 60 ± 19 Gt yr^−1^ and 57 ± 25 Gt yr^−1^ in the 1980s and 1990s, respectively. Ice losses then accelerated, reaching a rate three times higher in the 2000s, at 192 ± 19 Gt yr^−1^. In the 2010s, the rate of Greenland mass loss reached 264 ± 18 Gt yr^−1^ and was the first decade for which ice losses from reduced SMB exceeded ice losses from ice dynamical processes. During the period 2020 to 2023, while dynamic ice losses continued at a similar pace, the rate of SMB ice losses was only half that of the 2010s, resulting in a rate of Greenland mass loss of 199 ± 29 Gt yr^−1^. Unlike the 2010s which were marked by several extreme summer melt events leading to record mass loss events, there was no such extreme events in the past four years of our record, resulting in SMB-driven mass loss returning to a similar level to that of the 2000s. In total, Greenland lost 6,215 ± 467 Gt of ice at an average rate of 119 ± 9 Gt yr^−1^ between 1972 and 2023, 67% of which were caused by increased ice discharge and 33% by reduced SMB. Combined, Antarctica and Greenland lost 11,309 ± 565 Gt of ice between 1979 and 2023, raising the global sea level by 31.4 ± 1.6 mm. Increased glacier dynamical imbalance drove 84% of the ice sheets mass loss and reduced SMB the remainder (16%).

**Table 4:** Rates of ice sheet mass balance (MB), surface mass balance anomaly (SMB), and ice dynamics anomaly (D) (Gt yr^−1^).

		1980–1989	1990–1999	2000–2009	2010–2019	2020–2023	1972–2023	1979–2023
**GrIS (Gt yr**^**−1**^**)**	**MB**	−60 ± 19	−57 ± 25	−192 ± 19	−264 ± 18	−199 ± 29	−120 ± 9	−146 ± 10
	**SMB**	−5 ± 17	−10 ± 19	−73 ± 20	−141 ± 24	−76 ± 43	−40 ± 9	−58 ± 10
	**D**	−55 ± 26	−47 ± 31	−119 ± 28	−123 ± 30	−123 ± 52	−80 ± 13	−88 ± 14
**AIS (Gt yr**^**−1**^**)**	**MB**	−48 ± 13	−63 ± 20	−136 ± 22	−202 ± 22	−59 ± 73	—	−106 ± 10
	**SMB**	18 ± 13	−23 ± 13	15 ± 13	−32 ± 13	241 ± 14	—	18 ± 6
	**D**	−66 ± 19	−40 ± 24	−151 ± 25	−170 ± 26	−300 ± 75	—	−124 ± 12
**APIS (Gt yr**^−1^**)**	**MB**	−12 ± 4	−8 ± 5	−32 ± 5	−33 ± 5	11 ± 10	—	−18 ± 2
	**SMB**	−1 ± 2	−2 ± 2	2 ± 2	5 ± 2	22 ± 2	—	1 ± 1
	**D**	−11 ± 4	−6 ± 6	−34 ± 5	−38 ± 5	−11 ± 11	—	−19 ± 3
**EAIS (Gt yr**^−1^**)**	**MB**	−8 ± 11	−9 ± 16	−9 ± 18	−6 ± 18	92 ± 63	—	1 ± 9
	**SMB**	17 ± 7	−14 ± 7	9 ± 7	−26 ± 7	122 ± 9	—	9 ± 3
	**D**	−24 ± 13	5 ± 18	−17 ± 20	20 ± 20	−30 ± 64	—	−9 ± 9
**WAIS (Gt yr**^−1^**)**	**MB**	−29 ± 7	−45 ± 11	−96 ± 10	−163 ± 12	−162 ± 34	—	−89 ± 5
	**SMB**	3 ± 3	−4 ± 3	7 ± 3	−7 ± 3	53 ± 4	—	6 ± 2
	**D**	−31 ± 7	−41 ± 11	−103 ± 11	−156 ± 12	−215 ± 34	—	−95 ± 5

Rates are calculated from the first day (1^st^ January) of the first year quoted to the last day (31^st^ December) of the final year quoted in the table.

## Technical Validation

### Comparison within the input-output, altimetry, and gravimetry groups

**Fig. 3:**
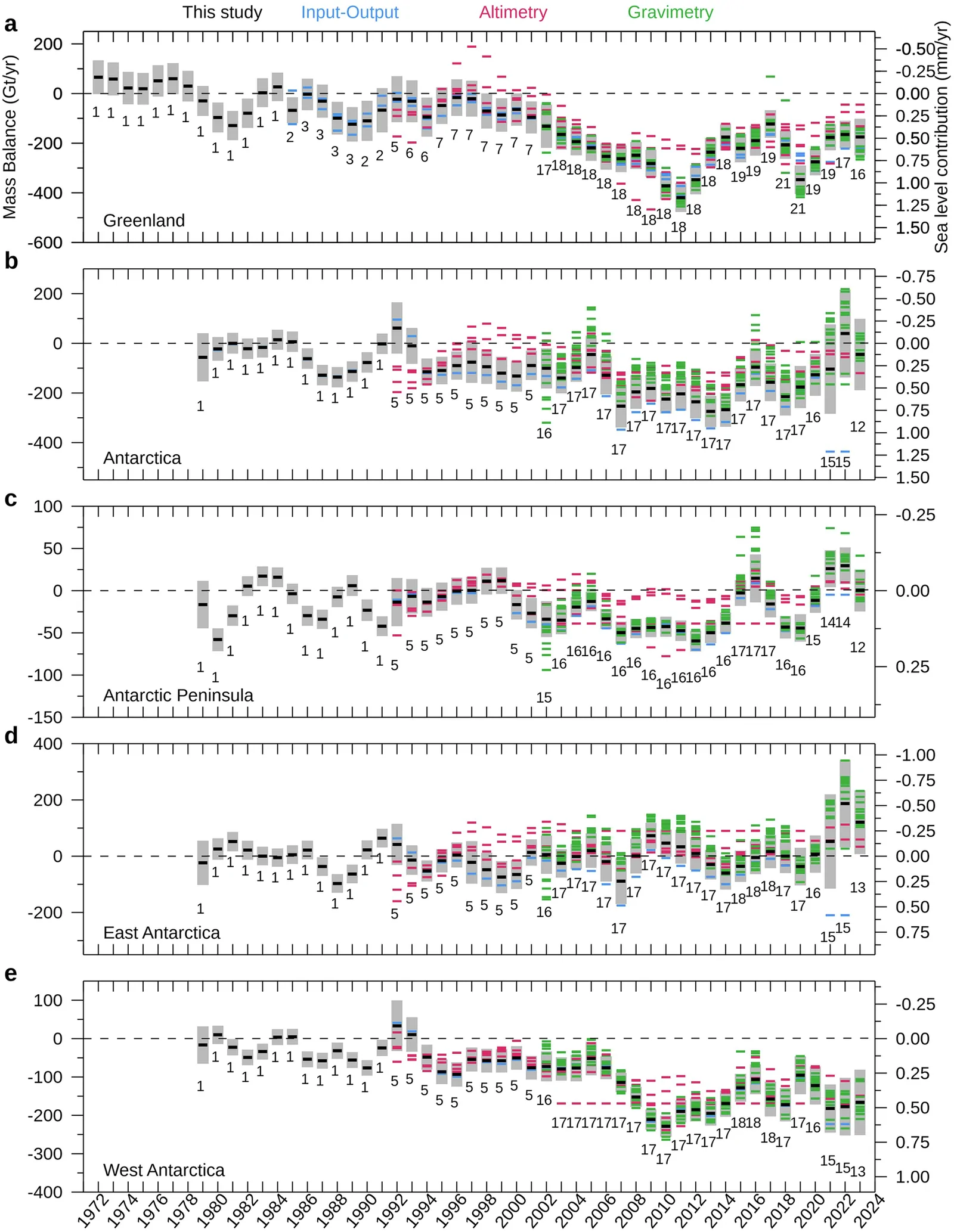
Annual rates of mass change of the (a) GrIS, (b) AIS, (c) APIS, (d) EAIS, and (e) WAIS from the altimetry, gravimetry and input-output estimates included in this study (shown by the coloured bars) and the reconciled estimate produced from combining those estimates (shown by the thick black bars). The grey shading shows the uncertainty of our final reconciled estimate. The number of individual mass balance estimates collated at each epoch is shown below each bar. For Greenland, the individual time-series that did not originally include the GICs have been corrected.

We assess the level of agreement among ice sheet mass balance estimates derived using the same technique by comparing annual rates of mass balance and their standard deviation for each ice sheet region (Fig. 3). In Greenland, input-output estimates are in good agreement with a median difference of 21 Gt yr^−1^ and standard deviation of 48 Gt yr^−1^ during their overlap period (1986–2021). Gravimetry estimates reach a slightly higher level of agreement during their overlap period (2018–2022) with a median difference 20 Gt yr^−1^ and standard deviation of 43 Gt yr^−1^. Finally, altimetry solutions, because of their varying temporal resolution (from 1 month to 16 years) present larger differences with a median difference of 64 Gt yr^−1^ and standard deviation of 51 Gt yr^−1^ when compared over the period 2011–2018 (and excluding the shortest time-series that spans only two years of data to allow for a more comprehensive comparison). In Antarctica, only one input-output estimate is available and thus no comparison with similar estimates can be performed. Differences between gravimetry estimates during their overlap period (2015–2022) are greatest in EAIS and smallest in APIS followed by WAIS with median differences of 31, 7, and 23 Gt yr^−1^ and standard deviations of 45, 11, 23 Gt yr^−1^, respectively. Similarly, altimetry estimates also exhibit the largest disagreement in EAIS, followed by WAIS and APIS with median differences of 42, 37, and 22 Gt yr^−1^ and standard deviations of 39, 36 and 20 Gt yr^−1^ over the period 2003–2018, respectively. Overall, we find that the vast majority of individual estimates of annual rates of mass balance included in this study fall within the uncertainty bounds of our reconciled estimate given their respective individual errors, with 93%, 88%, 87%, 90%, and 94% of those annual mass change rates falling within the reconciled uncertainty range at the GrIS, AIS, APIS, EAIS, and WAIS, respectively.

### Comparison across the input-output, altimetry, and gravimetry groups

**Fig. 4:**
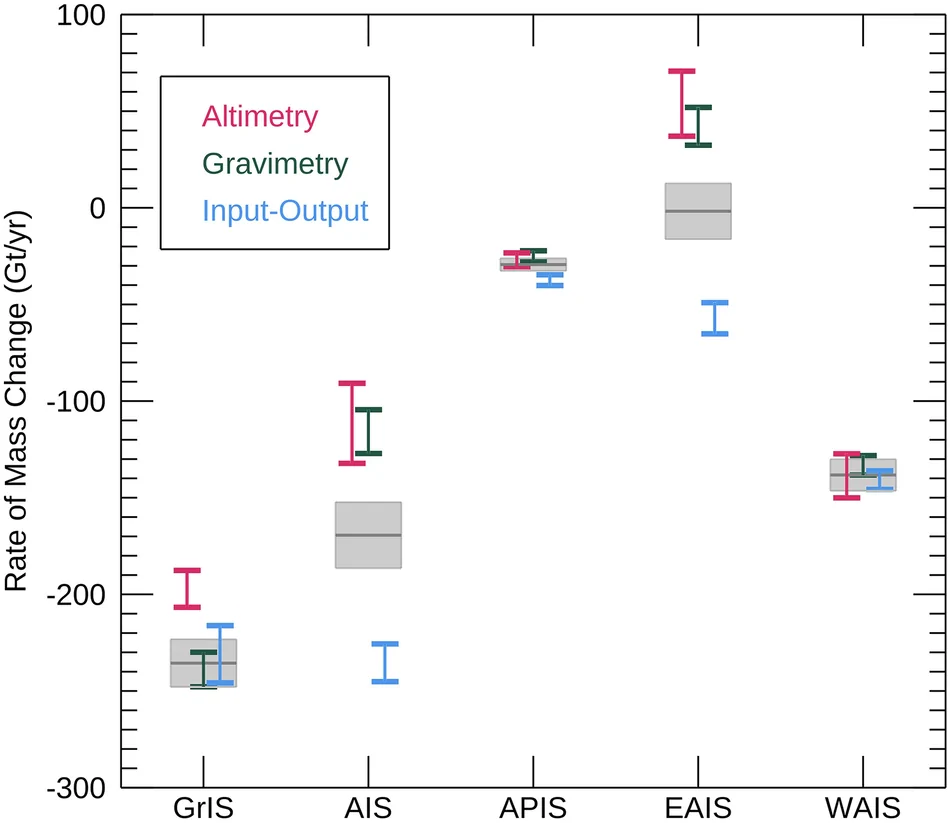
Inter-comparison of rates of ice sheet mass balance of the GrIS, AIS, WAIS, EAIS, and the APIS over the overlap period 2002–2023 for the GrIS and 2002–2021 for AIS and its sub-ice sheets from the altimetry, gravimetry, and input-output techniques. The coloured bars represent the rates of mass balance and uncertainties of the aggregated technique time-series. The grey box represents the rate of mass balance of our final reconciled assessment, with the horizontal line in the middle of the box showing the reconciled rate of mass balance and the height of the box representing its associated uncertainty.

We compare our three technique-aggregated input-output, altimetry, and gravimetry mass trends time-series during their overlapping period – over the period 2002–2023 for the GrIS and 2002–2021 for the AIS and its sub-ice sheets (Fig. 4). In Greenland, all three techniques are in close agreement with a standard deviation of 24 Gt yr^−1^ and a reconciled rate of mass loss of 236 ± 12 Gt yr^−1^. In Antarctica, the reconciled rate of mass loss is 169 ± 17 Gt yr^−1^ but the spread of the altimetry, gravimetry and input-output estimates is three times larger (70 Gt yr^−1^) than in Greenland. While at the WAIS and APIS the standard deviation between techniques is only 4 Gt yr^−1^ and 7 Gt yr^−1^, respectively, estimates differ significantly at the EAIS with a standard deviation of 61 Gt yr^−1^. At the EAIS, the three geodetic techniques disagree on even the sign of the mass change, with a maximum difference of 111 ± 19 Gt yr^−1^ between rates of mass change from the input-output method and altimetry estimates. We also note a substantial disagreement between the input-output group (−210 ± 73 Gt yr^−1^) and the altimetry and gravimetry groups (99 ± 124 Gt yr^−1^ and 166 ± 50 Gt yr^−1^) for 2021 calendar year, the last overlap year between the three techniques at the EAIS. Our assessment of Antarctica’s mass balance includes only one input-output estimate, which leads to an over-weighting of this estimate in our reconciled estimate in comparison to the other individual estimates derived from satellite altimetry and gravimetry, potentially introducing a small underestimation of mass gains in East Antarctica where the three techniques are in disagreement. To understand the departure of the input-output estimate in East Antarctica, we suggest that it is necessary to assess the availability of ice thickness measurements. Indeed, in regions of East Antarctica such as Enderby Land, Princess Elizabeth Land, Dronning Maud Land, and Wilkes Land, the availability of bed topography measurements in the vicinity of the grounding line, a key input for the computation of ice discharge, is very limited with over 60% of the grounding line of these regions lacking a measurement within 5 km of the actual grounding line location^45^. Surveying ice thickness around the grounding line in these regions and analysing differences between techniques at a regional scale would help understand the potential sources of the over-estimation of mass loss in East Antarctica in the input-output method.

### Comparison to previous IMBIE dataset

**Fig. 5:**
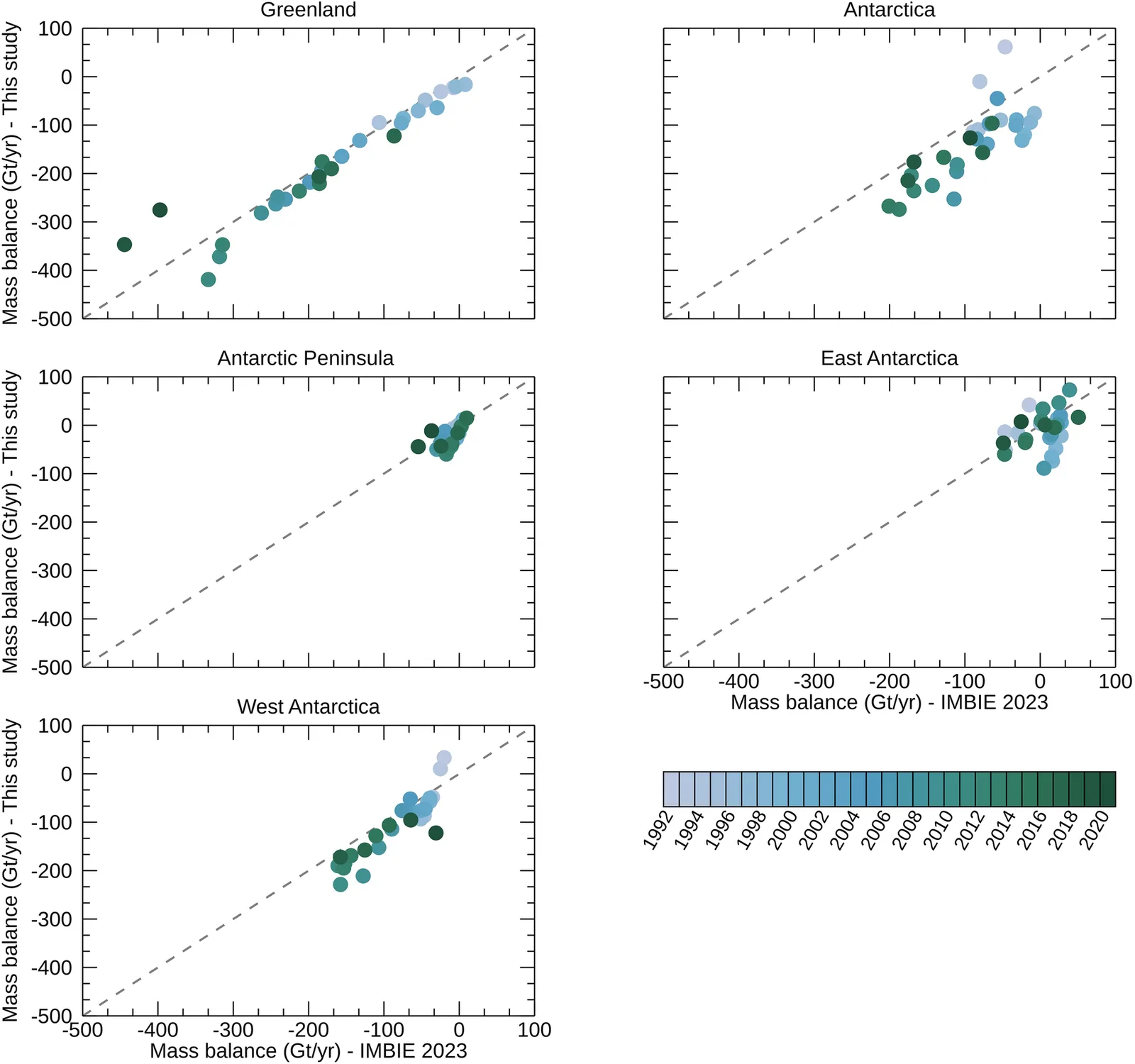
Comparison of rates of annual mass balance presented in This Study to a previous ice sheet mass balance assessment^2^ (IMBIE 2023).

We compare our dataset with the most recent consensus estimate of ice sheet mass balance^2^, produced as part of the Ice Sheet Mass Balance Inter-comparison Exercise (IMBIE) during their overlap period (1992–2020) (Fig. 5). In Greenland, the two datasets agree within their respective uncertainties with rates of mass loss of 180 ± 12 Gt yr^−1^ and 169 ± 16 Gt yr^−1^, respectively. In Antarctica, our dataset indicates larger rates of mass loss compared to the previous IMBIE assessment with rates of 140 ± 13 Gt yr^−1^ and 92 ± 18 Gt yr^−1^, respectively. When comparing annual rates of mass balance, we find that in Greenland the two datasets also further agree within their respective uncertainties in every year of their overlapping period and differences between the two datasets are only statistically significant at the AIS and APIS with corresponding p-values of 0.01 and 0.03, respectively. Differences between the two datasets exceed uncertainty bounds in four individual years at the APIS (2010, 2012, 2013, and 2014), in three years at the WAIS (2009, 2010, and 2020) and in two years for the Antarctic ice sheet as a whole (2000, 2007). We further investigate the origins of these differences by comparing the three technique-aggregated mass balance time-series (altimetry, gravimetry, and input-output) from the two datasets. Only our altimetry-aggregated mass balance time-series at the APIS is statistically different from the previous IMBIE assessment with a p-value of 0.001, primarily because the individual altimetry time-series included in this assessment have been significantly updated, with now more teams providing mass changes at finer temporal resolutions. As a result, we record a higher altimetry-derived rate of mass loss at the APIS of 20 ± 3 Gt yr^−1^ between 1992 and 2018 compared to 6 ± 1 Gt yr^−1^ from the previous IMBIE assessment, bringing our altimetry-aggregated estimate closer to the gravimetry and input-output estimates, with all three estimates now in agreement at the APIS. The remaining small differences between our previous and current mass balance assessments at the GrIS, WAIS, and EAIS originate from the inclusion of new and updated participant datasets and in Greenland from the systematic inclusion of its peripheral GICs.

## Data Availability

The aggregated Greenland and Antarctic ice sheet mass balance data and corresponding errors are available at https://doi.org/10.5285/128c5e33-5224-4197-82f0-19dcc95b80a0.

## Code availability

The code used to compute and aggregate rates of ice sheet mass change and their errors is freely available at https://doi.org/10.5281/zenodo.14217192^46^.
